# “Listening to the word autism changes everything”: the impact of an Autism Spectrum disorder diagnosis on families

**DOI:** 10.3389/fpsyg.2026.1919815

**Published:** 2026-09-11

**Authors:** M Gloria Gallego-Jiménez, Sofía Torrecilla-Manresa, Cristina García-Bravo

**Affiliations:** 1 Faculty of Humanities and Communication, Department of Education. CEU San Pablo University, Madrid, Spain; 2 Department of Physical Therapy, Occupational Therapy, Rehabilitation and Physical Medicine, Research Group of Humanities and Qualitative Research in Health Science, Universidad Rey Juan Carlos, Alcorcon, Spain

**Keywords:** Autism Spectrum disorder, diagnosis, lived experience family life, parents, qualitative study

## Abstract

**Introduction:**

An autism spectrum disorder (ASD) diagnosis is a transformative event for families that extends beyond clinical identification and affects the emotional, social and organizational dimensions of everyday life. This descriptive exploratory qualitative study aimed to explore the lived experiences and expectations of Spanish parents during their child's ASD diagnostic process, as well as the impact of the diagnosis on family life and concerns about the future.

**Methods:**

Seventeen parents of children diagnosed with ASD participated in online semi-structured interviews. Participants received information about the study procedures, interview recording, confidentiality and data handling before providing written informed consent. Interviews were audio-recorded, transcribed verbatim and analyzed using inductive qualitative content analysis. Each interview was initially examined as a distinct experiential account situated within its family and social context before shared and divergent patterns were compared across participants.

**Results:**

Three overarching themes were identified: (1) the impact of diagnosis and the process of family adaptation; (2) the emotional impact, coping strategies and reorganization of family life; and (3) concerns about the future. Parents described a prolonged diagnostic process characterized by uncertainty, limited professional validation of their initial concerns and an ambivalent emotional response involving shock, sadness and relief. Grief and adjustment were experienced as non-linear and recurrent processes associated with the reformulation of previously imagined expectations. Parents described coping through peer support, the active search for services and personal assistance, the structuring and anticipation of everyday routines and the flexible adjustment of goals to their child's abilities. Although these strategies helped parents manage everyday demands, they also required sustained vigilance and considerable effort. The diagnosis additionally intensified the parent-child bond, affected siblings and substantially reorganized family life. Concerns about future autonomy, continuity of care and the availability of adult support remained central.

**Discussion:**

These findings highlight the need for clear post-diagnostic guidance, sustained emotional support and coordinated, family-centered services across the life course.

**HIGHLIGHTS**

- ASD diagnosis triggered uncertainty, grief, relief and family reorganization.
- Parents described a prolonged diagnostic odyssey before receiving answers.
- Post-diagnosis guidance was perceived as fragmented and insufficient.
- Parents became advocates while adapting expectations to neurodiversity.
- Future care, autonomy and adult support were central family concerns.

## 1 Introduction

Autism Spectrum Disorder (ASD) is a multifaceted neurobiological condition that is characterized by a wide variety of symptoms that profoundly affect communication, social interaction and behavior, with notable heterogeneity in terms of clinical expression and support needs (Sánchez Amate and Luque de la Rosa, 2024; World Health Organization (WHO), 2025). In recent decades, the increase in its prevalence has seen ASD has acquired increasing public health and social relevance. Global prevalence is estimated to be increasing, affecting approximately 0.77% of children worldwide (Issac et al., 2025). In Spain, this trend is evident in the significant increase in children diagnosed with ASD in the educational system, exceeding 90,000 students and showing sustained growth in recent years (Autismo España, 2026).

This increase not only reflects improvements in screening and diagnostic processes, but also greater social awareness and changes in diagnostic criteria (Centers for Disease Control Prevention (CDC), 2023). However, beyond the epidemiological aspect, an ASD diagnosis is a profoundly transformative event in the lives of families. Far from being limited to a mere clinical categorization, it signifies a rupture in the life stories of the parents, affecting their expectations, their identity and the organization of their daily lives (Boss, 1999; Woodgate et al., 2008; Dounavi and Koldas, 2025). While healthcare typically focuses on the child receiving the diagnosis, its implications extend to the wider family system, affecting interpersonal relationships and parents' emotional wellbeing (Sánchez Amate and Luque de la Rosa, 2024).

Although early detection is essential to ensure timely interventions, clinical reality shows that the period that elapses between the first parental suspicions and the formal diagnosis of neurodevelopmental disorders is often excessively long (Daniels et al., 2021; Dounavi and Koldas, 2025). The literature describes this process as a “biographical disruption,” in which families go from an initial phase of uncertainty, marked by the identification of early signs, to diagnostic confirmation and a subsequent reorganization of their lives (Edwards et al., 2018; García-Bravo et al., 2023; Dounavi and Koldas, 2025). Throughout this journey, parents encounter numerous structural barriers, including diagnostic delays, limited institutional guidance and difficulties in accessing specialized resources, contributing to what has been described as a “diagnostic odyssey” (Creswell and Poth, 2018; Centers for Disease Control Prevention (CDC), 2023, 2025). In this study, health and social-care support refers to the coordinated healthcare, educational, community and social services accessed by families during the diagnostic process, throughout childhood and during the transition to adulthood.

Parents in previous studies have described the process as a “tortuous path” in which, faced with the minimization of the first symptoms by some health professionals (especially in primary care), they are forced to wage a constant struggle to be heard and find answers (Garcia-Bravo et al., 2025). In Spain, these difficulties are exacerbated by delays in accessing diagnostic tests and the limited availability of resources within the public healthcare system, forcing many families to turn to private healthcare and bear the associated costs All of this leads to high levels of stress and emotional strain, with direct repercussions on the psychological wellbeing of parents and, indirectly, on the child's development (Sánchez Amate and Luque de la Rosa, 2024).

Caregiving should not be understood as beginning at the point of diagnostic confirmation. Before a formal diagnosis, parents may already be engaged in close observation, repeated help-seeking and informal adaptations in response to developmental differences that remain unexplained. Diagnostic confirmation may therefore function less as the origin of caregiving change than as a point of reframing: it can validate previous concerns, reorganize expectations and redirect parental efforts toward formal interventions, service navigation, advocacy and long-term planning (Edwards et al., 2018; García-Bravo et al., 2023; Dounavi and Koldas, 2025). Caregiving trajectories should consequently be understood as evolving across the pre-diagnostic, diagnostic and post-diagnostic periods rather than as a simple binary distinction between before and after diagnosis.

The emotional impact of receiving a diagnosis of a lifelong neurodevelopmental condition is not limited to an immediate or one-off reaction. Rather, parents may experience a dynamic process of grief and adjustment involving shock, disbelief, uncertainty, sadness, guilt, searching for explanations and, in some cases, relief after obtaining a framework through which to understand their child's development (Edwards et al., 2018; García-Bravo et al., 2023). These emotional responses should not be interpreted as fixed, universal or strictly sequential stages, as parents may move between them, experience several simultaneously or revisit them at different points in their child's development. In the context of ASD, this process has been linked to ambiguous or non-finite grief, referring not to the loss of the child, but to the transformation of previously imagined expectations regarding the child's development, family life and the future (Boss, 1999; O'Brien, 2007; Bravo-Benítez et al., 2019; Mihandoust et al., 2021). Feelings of loss and uncertainty may re-emerge during developmental transitions, comparisons with peers, unmet expectations or concerns about future autonomy and support, resembling chronic sorrow and reflecting the recurrent nature of parental adjustment (Coughlin and Sethares, 2017).

Concurrently, ASD may lead to substantial changes in family dynamics, including the redistribution of roles, the reorganization of everyday routines, an intensification of the parent–child bond, effects on siblings and, in some cases, social isolation associated with stigma (Sánchez Amate and Luque de la Rosa, 2024; Garcia-Bravo et al., 2025). Although parents may initially feel overwhelmed and insufficiently prepared to make decisions about treatments and interventions, they can gradually develop experiential knowledge and move from a conventional parenting role toward becoming advocates and experts in their child's needs (Edwards et al., 2018). This transition does not necessarily represent the resolution of grief, but rather an evolving process of integrating the diagnosis into family life while continually adapting expectations and caregiving strategies.

Family adjustment following an ASD diagnosis is also shaped by the strategies parents use to manage its emotional, practical and relational demands. In this context, coping includes behavioral, relational and meaning-oriented responses, such as seeking information and social support, reorganizing everyday routines, advocating for the child and reformulating previous expectations. Adaptive strategies, particularly social support and positive reappraisal, have been associated with better caregiver well-being and lower emotional distress (Sánchez Amate and Luque de la Rosa, 2024). Nevertheless, coping strategies are context-dependent, and their usefulness may vary according to family circumstances, available resources and the child's support needs.

From a family systems perspective, the family is understood as an interdependent relational system in which changes affecting one member influence the functioning, roles and relationships of the family as a whole (Cox and Paley, 1997; Rogers et al., 2022). Family subsystems interact bidirectionally and continually adapt to changes occurring both within the household and in the wider social environment (Rogers et al., 2022). An ASD diagnosis may therefore require adjustments across parent–child and sibling relationships, including the redistribution of care responsibilities, the reorganization of time and attention and the renegotiation of previously established roles and boundaries. Parents may move beyond conventional parenting roles to act as advocates, coordinators and interpreters of their child's needs, while siblings may experience changes in parental attention, family expectations and anticipated future responsibilities. These adjustments also occur alongside a confrontation between previously imagined family trajectories and the child's actual developmental and support needs.

Alongside the demands of daily life, concerns about the future are a central feature of families' experiences, particularly with regard to the child's independence, the availability of support in adulthood and the continuity of care (Garcia-Bravo et al., 2025; Autismo España, 2024). These concerns are exacerbated by the perception that resources are reduced once the educational phase is over, highlighting the need for an in-depth understanding of family experiences from a holistic perspective (Autismo España, 2024; Issac et al., 2025).

Qualitative research is particularly valuable for understanding complex processes from the perspectives of those who experience them and for uncovering the beliefs, values and motivations underlying individual health-related behaviors (Carpenter and Suto, 2008; Moser and Korstjens, 2017; Korstjens and Moser, 2017). Within qualitative research, descriptive exploratory studies provide an opportunity to investigate experiences of illness and disability in real-life contexts, incorporating individuals' perspectives within their own social environments (Bradshaw et al., 2017; Jack and Phoenix, 2022). In this study, lived experience is understood as the subjective and context-dependent way in which parents perceive, interpret and give meaning to their child's diagnostic process and its consequences for everyday family life (Neubauer et al., 2019). These experiences are shaped by the child's characteristics and support needs, family composition, parents' previous expectations, available resources and interactions with healthcare, educational, community and social-care services. Consequently, the diagnostic process is not assumed to follow a single or uniform trajectory. Rather, each parental account is understood as a distinct experiential account situated within a particular family and social context. This perspective enables the uniqueness of each parent's situated experience to be preserved while allowing shared and divergent patterns across participants' accounts to be identified.

Despite the growing interest in ASD, further qualitative research is needed to explore parents' lived experiences in depth while recognizing variation across individual experiences and family contexts. Understanding these experiences is essential for informing family-centered interventions, public policies and models of care.

The aim of this study was to explore the lived experiences and expectations of Spanish parents of children with ASD during the diagnostic process, as well as their perceptions of the impact of diagnosis on everyday family life, family relationships and concerns about the future.

## 2 Materials and methods

### 2.1 Study design

A descriptive qualitative study with an exploratory orientation was conducted, following established methodological frameworks (Jack and Phoenix, 2022; Bradshaw et al., 2017). The study was conducted in accordance with international reporting standards for qualitative research, namely the COREQ (Tong et al., 2007) and SRQR (O'Brien et al., 2014) guidelines, as promoted by the EQUATOR Network.

Qualitative methodologies are particularly well suited to examining complex phenomena from the perspectives of those directly involved, enabling an in-depth exploration of the meanings, beliefs and contextual factors associated with health-related experiences (Carpenter and Suto, 2008; Moser and Korstjens, 2017). Within this framework, a descriptive exploratory approach facilitated the examination of experiences related to disability and the diagnostic process in real-life contexts while considering participants' social environments (Bradshaw et al., 2017; Jack and Phoenix, 2022). This study focused on the expectations and lived experiences of Spanish parents of children with ASD throughout the diagnostic process and on their perceptions of the consequences of diagnosis for everyday family life. The study was informed by a lived-experience perspective, which recognizes each parent's account as subjective, context-dependent and situated within a particular family and social environment. Accordingly, the focus was on individual parental accounts rather than on dyadic or whole-family narratives.

### 2.2 . Participants

#### 2.2.1 Sampling strategy and sample size

Participant selection was conducted using purposive sampling, targeting individuals with direct and relevant experience related to the study's aims (Creswell and Poth, 2018; Carpenter and Suto, 2008). To facilitate access to the sample, snowball sampling was also used, with initial participants identifying other potential candidates.

In qualitative research, sample size was determined by data saturation rather than statistical criteria of representativeness (Creswell and Poth, 2018; Moser and Korstjens, 2018). Empirical studies suggest that saturation is typically reached between nine and 17 interviews and can be extended to 15–23 in more structured designs (Hennink and Kaiser, 2022; Squire et al., 2024).

#### 2.2.2 Inclusion and exclusion criteria

Selection criteria were established to ensure the sample's suitability for the phenomenon under study. Participants were included if they: (a) were parents of children with a clinical diagnosis of ASD, (b) had children enrolled in schools with an inclusive approach in Spain, (c) had recent and ongoing experience with the education system (at least during the last academic year) and (d) had sufficient proficiency in Spanish to be able to participate in interviews and provide informed consent.

Participants were excluded if they: (a) did not actively participate in their children's education, (b) faced barriers that prevented their participation or (c) did not provide or withdrew informed consent at any stage of the study.

#### 2.2.3 Recruitment

A recruitment notice was circulated through collaborating autism associations and schools with an inclusive educational approach. The notice briefly described the study purpose, eligibility criteria, voluntary nature of participation, interview format and contact details of the research team. Staff from the collaborating organizations did not disclose the identities or contact details of potential participants to the researchers.

Parents who were interested in participating contacted the research team directly and were invited to an initial information session, during which the study objectives, procedures and participation requirements were explained. In addition, enrolled participants were invited to share the recruitment notice with other potentially eligible parents as part of the snowball sampling strategy. These individuals were also required to contact the research team directly, and no third-party contact details were provided by participants.

Parents who agreed to participate received the participant information sheet and informed consent form by email. Once written informed consent had been obtained, the interview was scheduled and participants were provided with access details for the online session.

#### 2.2.4 Data gathering

Data were collected through one individual semi-structured interview with each participating parent, resulting in 17 interviews. Interviews lasted between 40 and 80 minutes and were conducted online via Microsoft Teams, which allowed for a comprehensive exploration of the participants' experiences.

A semi-structured interview guide was developed for this study. Its thematic domains were informed by Gallego-Jiménez et al. (2025)) and adapted to the study aim and participant population. The guide used open-ended questions and flexible follow-up prompts to explore emerging aspects of participants' experiences. The core guiding questions and the areas explored are presented in Table 1. All interviews were conducted by two members of the research team (G.M.G.J. and S.T.-M.) university researchers with previous experience in semi-structured qualitative interviewing. The interviewer had no previous relationship with the participants.

**Table 1:** Interview topic guide.

Main theme	Areas explored	Core guiding question and possible follow-up questions
Diagnostic process	Initial concerns, interaction with professionals, diagnostic confirmation	Core guiding question: Can you tell me about the process from the moment you first became concerned about your child's development until the diagnosis was confirmed? Possible follow-up questions: What initially drew your attention? How were your concerns received by professionals? Were there any delays, difficulties or turning points during the process?
Impact of diagnosis	Emotional response, meaning of the diagnosis, changes in expectations	Core guiding question: How did you experience the moment when the diagnosis was communicated, and what did the diagnosis mean to you at that time? Possible follow-up questions: What thoughts or emotions did you experience? Did receiving the diagnosis change how you understood your child's development? Did it influence your expectations regarding your child or family life?
Guidance and support	Information received, access to resources, institutional support	Core guiding question: What information, guidance or support did you receive during and after the diagnostic process? Possible follow-up questions: Did you know what steps to take after the diagnosis? Which professionals, organizations or other parents helped you? What information or support did you feel was missing?
Family adaptation	Coping strategies, acceptance, evolution of the parental role	Core guiding question: How have you and your household adapted to your child's needs throughout the diagnostic process? Possible follow-up questions: Has your role as a parent changed? What strategies have helped you cope or adapt? Have your priorities, goals or expectations changed over time?
Daily life	Routines, daily organization, lifestyle changes	Core guiding question: Can you describe how everyday family life is currently organized around your child's needs? Possible follow-up questions: What role do routines and planning play? How do you manage unexpected situations or changes? Have outings, leisure activities or social relationships been affected?
Family dynamics	Parent–child relationship, impact on siblings, family relationships	Core guiding question: How do you perceive that your child's needs and the diagnostic process have influenced relationships and responsibilities within your household? Possible follow-up questions: Has your relationship with your child changed? How are time, attention and responsibilities distributed? How do you think siblings or other family members experience the situation?
Emotional burden	Stress, associated emotions, psychological well-being	Core guiding question: How has this experience affected you emotionally over time? Possible follow-up questions: Have your emotional responses changed since the first concerns or the diagnosis? Are there feelings or concerns that reappear at particular moments? What situations are currently most emotionally demanding?
Future perspectives	Autonomy, long-term concerns	Core guiding question: What are your main hopes and concerns regarding your child's future? Possible follow-up questions: What does independence mean in your child's particular situation? What concerns you about future care? How do you imagine your child's life during adolescence and adulthood?
Support systems	Resources in adulthood, social and employment inclusion	Core guiding question: What forms of support does your child currently receive, and what support do you think will be needed in the future? Possible follow-up questions: How would you describe the coordination between services? What concerns do you have about the end of compulsory education? What resources, employment opportunities or community support do you think are currently missing?
Overall reflections	Learning experiences and perceived needs	Core guiding question: Looking back on the process, what have you learned and what do you think professionals or services should do differently? Possible follow-up questions: What would have helped you during the diagnostic process? What advice would you give to professionals supporting other parents? Is there anything important that we have not discussed?

Source: Prepared by author.

The interview-guide domains were used to facilitate a broad and systematic exploration of experiences relevant to the study aim. They informed data generation but were not intended to constitute predetermined analytical categories. The semi-structured format also allowed participants to introduce relevant experiences across different domains and enabled the interviewer to adapt the order and depth of the questions to each account.

During and immediately after each interview, the interviewer recorded field notes concerning the interview context, relevant emotional or non-verbal expressions observable during the online session, interruptions or technical incidents and initial observations related to the participant's account. These notes were used to contextualize the transcripts during analysis (Carpenter and Suto, 2008).

In addition, members of the analysis team maintained reflexive journals throughout the coding and theme-development process. These journals documented researchers' assumptions, reactions to the data, analytical decisions, coding disagreements and changes made during category and theme development. Reflexive notes were reviewed during research-team meetings to examine the potential influence of the researchers' perspectives and to support transparent analytical decision-making. They were used as contextual and reflexive material rather than as primary participant data (Carpenter and Suto, 2008).

Any subsequent contact conducted for member checking was limited to confirming or clarifying the information obtained and was not treated as an additional research interview.

Sociodemographic data were collected from the participating parents, including age, gender, occupation and number of children. Parents also provided information about their child with ASD, including age, gender and reported level of support. The year or date of diagnosis was not collected as a standardized participant characteristic.

#### 2.2.5 Data management and confidentiality

Before providing informed consent, participants received written and oral information about the purpose of the study, the voluntary nature of participation, the audio-recording of the interviews, the use of anonymised quotations in scientific publications, the procedures used to protect confidentiality, the storage and retention of the data, and their right to withdraw from the study without providing a reason. Participants were informed that they could withdraw their data until the transcripts had been anonymised and incorporated into the aggregated analysis.

Each participating parent was assigned an alphanumeric identification code ranging from P1 to P17. These codes referred to individual participants rather than to family units. Identifying information was stored separately from the interview data. Audio recordings were transferred immediately after each interview to an encrypted, password-protected institutional server and were deleted from Microsoft Teams. Recordings, pseudonymised transcripts, field notes and ATLAS.ti files were accessible only to the research team. Any quotations included in publications were reviewed to remove names and potentially identifying contextual information.

#### 2.2.6 . Data analysis

An inductive qualitative content analysis was conducted in accordance with Graneheim and Lundman (2004)), Graneheim et al. (2017)) and Lindgren et al. (2020)). The analysis focused primarily on manifest content, including the experiences, events, concerns and meanings explicitly expressed by participants. During category and theme development, the researchers also considered latent content, understood as the underlying meanings, tensions and patterns conveyed across participants' accounts. This interpretive step was applied at the level of categories and themes and remained grounded in the wording and context of each interview.

Consistent with the inductive approach, the domains included in the interview guide were not used as a deductive coding framework. Codes, categories and themes were developed from the interview data through open coding, comparison and abstraction. Consequently, material elicited under one interview domain could contribute to more than one analytical category or theme, while conceptually related material arising from different domains could converge within the same theme (Graneheim and Lundman, 2004; Elo and Kyngäs, 2008; Hsieh and Shannon, 2005; Lindgren et al., 2020).

The individual parent was the unit of participation and analysis. Each interview was initially analyzed as an individual parental account situated within its specific family and social context before shared and divergent patterns were compared across participants' accounts. No dyadic or whole-family analysis was undertaken. The year or date of diagnosis was not collected as a standardized participant characteristic; therefore, time since diagnosis was not available for sampling, stratification or comparative analysis. The study was cross-sectional and was not designed to examine changes longitudinally.

Although participants' accounts included retrospective references to pre-diagnostic concerns, diagnostic confirmation, subsequent adaptation and future-oriented concerns, these references arose within the interview narratives and were treated as contextual elements within each individual account rather than as evidence of a single chronological trajectory shared by all participants. Qualitative longitudinal research designed to examine change over time explicitly incorporates temporality into the research aims, sampling, data collection and analytical strategy and commonly involves repeated data collection across several time points, which was not the case in the present study (Audulv et al., 2022). Consistent with inductive qualitative content analysis, the final analytical structure therefore prioritized conceptually coherent patterns across accounts rather than imposing a binary pre-diagnostic versus post-diagnostic framework on the data (Graneheim et al., 2017; Lindgren et al., 2020).

The interviews were transcribed verbatim. Meaning units were identified, condensed and assigned descriptive codes. Codes were then compared and grouped into categories according to their conceptual similarity. During theme development, the researchers examined the broader meanings and tensions connecting these categories while preserving their relationship with the original accounts.

Consistent with inductive qualitative content analysis, themes and subthemes were developed on the basis of the meaning, context and analytical relevance of the coded material rather than through the numerical ranking of codes or quotations. Recurrence across interviews was considered as one indication that an experience was shared, but no predetermined frequency threshold was applied to determine the validity or importance of a theme or subtheme. Candidate subthemes were retained when they were conceptually coherent, relevant to the study aim, distinguishable from adjacent subthemes and traceable to the original meaning units. Less frequent accounts could also be retained when they represented meaningful variation or contributed to a more comprehensive understanding of parents' experiences (Hsieh and Shannon, 2005; Elo and Kyngäs, 2008; Graneheim et al., 2017; Lindgren et al., 2020; Sandelowski, 2001).

Two researchers independently coded the interview transcripts and compared their initial coding. A third researcher reviewed the shared analytical matrix and participated in discussions concerning coding discrepancies, category development and theme development. Differences were resolved through discussion and consensus among the three researchers. Regular analytical meetings were held to document decisions and refine the categories and themes. ATLAS.ti software (version 26) was used to organize the transcripts, assign and retrieve codes, group related codes and preserve traceable links between coded meaning units and their original contexts. The software supported data organization, retrieval and documentation but did not replace the researchers' interpretive work or generate categories and themes automatically (Vignato et al., 2022). All coding decisions, category development and theme construction were undertaken by the research team through iterative comparison, reflexive discussion and consensus.

Interview transcripts constituted the primary participant data. Field notes were reviewed alongside the corresponding transcripts to contextualize the interview conditions, relevant emotional expressions observable during the online session, interruptions and other contextual elements that could inform the interpretation of participants' accounts. When the field notes provided additional context or suggested alternative interpretations, these observations were discussed during research-team meetings.

Reflexive journals were not analyzed as participant data. Instead, they were used to document researchers' assumptions, reactions to the data, coding decisions, disagreements and changes made during category and theme development. These records supported reflexive discussion and contributed to the audit trail of the analytical process.

Illustrative quotations were selected according to their semantic completeness, contextual clarity and relevance to the analytical interpretation rather than according to length alone. Consistent with qualitative content analysis, a meaning unit may comprise words, sentences or paragraphs connected through their content and context. Short quotations were therefore retained when they constituted self-contained semantic units and were clearly situated within the accompanying analytical narrative. Longer excerpts were used when additional context was required to represent more complex experiences or clarify the referent, temporal orientation or intended meaning of participants' accounts (Graneheim and Lundman, 2004; Eldh et al., 2020).

Quotations were translated from Spanish into English and checked against the original transcripts by researchers to preserve their semantic meaning.

#### 2.2.7 Criteria of rigour

Methodological rigour was addressed according to Lincoln and Guba's (1985)) criteria of credibility, transferability, dependability and confirmability. Credibility was supported primarily through researcher triangulation. Three researchers participated in the analytical process, compared coding and discussed divergent interpretations during regular meetings until consensus was reached.

Interview transcripts constituted the primary participant data, while field notes were used as complementary contextual material to support and refine the interpretation of individual accounts. As all participant data were generated through semi-structured interviews, this procedure was considered triangulation of researchers and integration of complementary contextual materials rather than full methodological triangulation. Member checking was also used to confirm or clarify information obtained during the interviews.

Transferability was supported through detailed descriptions of the study context, participants, sampling strategy, data collection and analytical procedures. Dependability was strengthened through an external audit conducted by a researcher who was not involved in the initial coding process. The audit examined the research protocol, analytical procedures and development of categories and themes.

Confirmability was supported through reflexive journals documenting researchers' assumptions, responses to the data and analytical decisions. These records were reviewed during team meetings to examine the possible influence of researchers' perspectives on coding and theme development. The strategies used to support methodological rigour are presented in Table 2 (Korstjens and Moser, 2018).

**Table 2:** Criteria of rigor.

Criteria	Techniques performed
Credibility	**Researcher triangulation:** Three researchers participated in the analytical process, compared coding and discussed divergent interpretations until consensus was reached. **Integration of contextual materials:** Field notes were reviewed alongside the corresponding interview transcripts to contextualize and refine interpretations. **Member checking:** Participants were contacted to confirm or clarify information obtained during the interviews.
Transferability	Detailed descriptions were provided of the study context, participants, sampling strategy, data collection procedures and analytical process.
Dependability	An external researcher who was not involved in the initial coding reviewed the research protocol, analytical procedures and development of categories and themes.
Confirmability	Reflexive journals were used to document researchers' assumptions, responses to the data, coding decisions and changes made during category and theme development. These records were reviewed during research-team meetings.

Source: prepared by author

The study was conducted in accordance with the Declaration of Helsinki and was approved by the Ethics Committee of CEU San Pablo University (code: 401.998). Written informed consent was obtained from all participants before inclusion. The information provided before consent explained the recording procedure, confidentiality safeguards, pseudonymisation, data storage, restricted access, intended use of anonymised quotations and the right to withdraw.

## 3 Results

A total of 17 parents of children diagnosed with ASD were recruited for this study. The sample was predominantly composed of mothers (88.24%), while fathers represented 11.76%. The age of the participants ranged from 32 to 51, with an average age of 45.5. A considerable degree of heterogeneity was observed in terms of educational attainment: approximately 65% held a university degree, around 25% had postgraduate studies and the remaining 10% had completed vocational training or secondary education.

As far as professional experience was concerned, 55% of the participants worked in the educational, social, or healthcare sectors, while the other 45% worked in administrative, commercial, technological, and service sectors. Regarding work experience, 40% said that they had more than 15 years of experience, 35% between 5 and 15 years and 25% less than 5 years, indicating a balanced representation of both established professionals and those in the early stages of their careers.

With respect to family composition, the majority of participants reported having 2 children (76.47%), with family sizes ranging from 1 to 3 children. The child with ASD most frequently occupied the second position in birth order (47.06%), although cases of only children and other positions within the sibling group were also identified. It is worth noting that, in two cases, the children with ASD were twins, both with a diagnosis.

Regarding gender distribution, males (73.33%) outnumbered females (26.67%) among children with ASD. Finally, according to the DSM-5 classification of support levels, 41.18% of the children were at level 1, while 29.41% were at level 2 and another 29.41% at level 3, suggesting a relatively balanced distribution across the levels indicating greater support needs.

Although the interview guide comprised ten broad domains, the inductive analysis generated three overarching themes. The interview domains supported comprehensive data generation but did not determine the analytical structure of the findings. Several domains converged within the same theme, and some domains contributed to more than one theme.

The findings are presented thematically rather than as a linear chronological sequence. Participants referred retrospectively to several temporal moments, including initial concerns, diagnostic confirmation, ongoing adaptation and future-oriented concerns. These temporal references arose from the narrative content of the interviews and were not linked to a standardized measure of time since diagnosis. Moreover, the moments described frequently overlapped and did not constitute a uniform trajectory shared by all participants. Temporal shifts are therefore identified within and across themes where they were supported by participants' accounts. This form of presentation was selected to preserve the inductively generated analytical structure and communicate the findings coherently rather than to reconstruct a pre-diagnostic and post-diagnostic comparison that was not part of the study design (Sandelowski, 1998).

**Figure 1:**
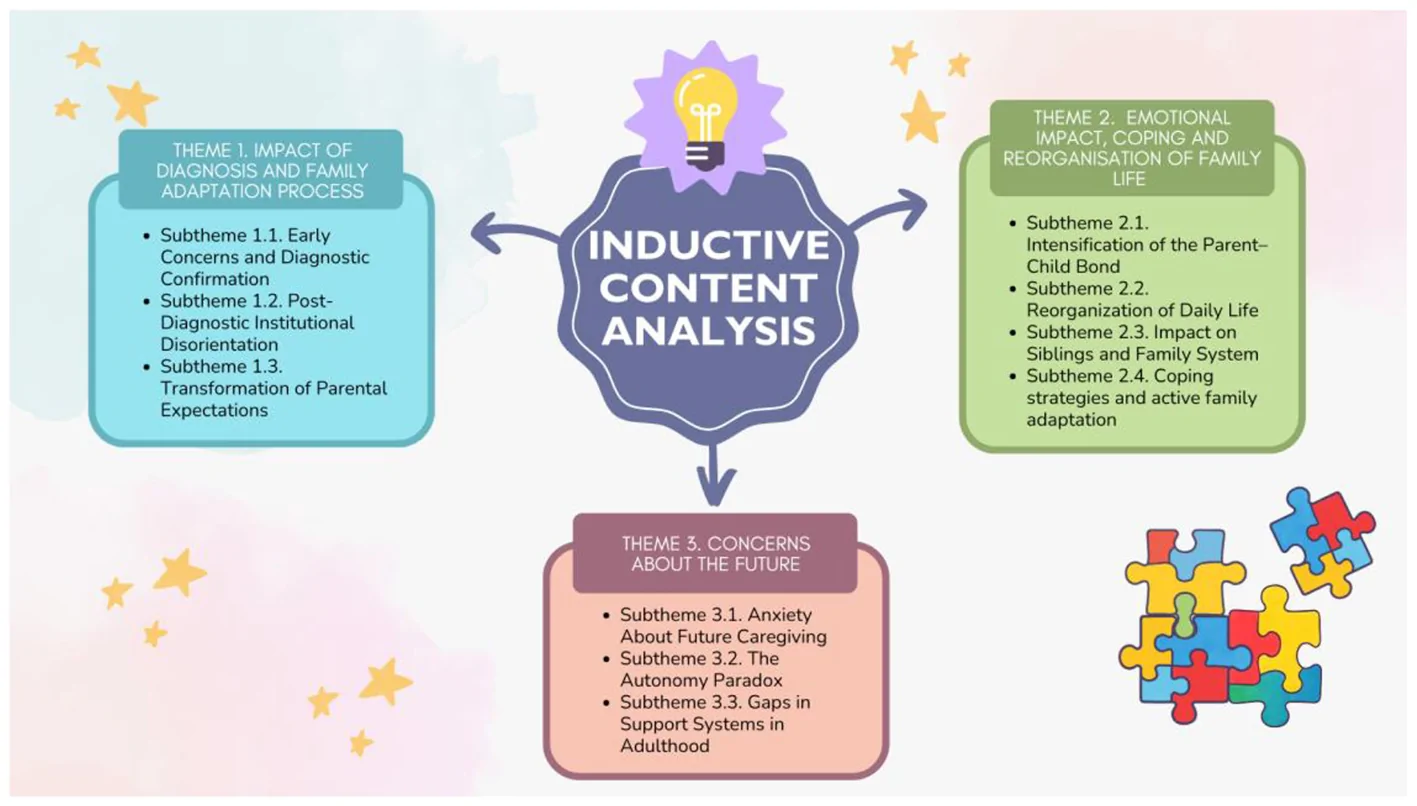
Hierarchical organization of the themes and subthemes generated through inductive qualitative content analysis.

Three main themes emerged from the analysis: (1) the impact of diagnosis and the process of family adaptation; (2) emotional impact, coping and reorganization of family life; and (3) concerns about the future (see Figure 1). The themes and subthemes represent conceptually coherent and analytically relevant patterns identified across participants' accounts. Their development was based on the meaning, context and relevance of the coded material rather than on numerical frequency alone. Direct quotations are provided as illustrative examples selected from the full coded dataset and do not represent the total number of meaning units supporting each subtheme. Quotations are identified using participant codes ranging from P1 to P17. These codes refer to individual parents and should not be interpreted as representing collectively constructed family accounts.

### 3.1 Theme 1: Impact of the diagnosis and the process of family adaptation

#### 3.1.1 Subtheme 1.1: Initial suspicions and diagnostic confirmation

Parents described an initial process marked by the detection of early signs that generated concern, such as a lack of response to their child's name or limited eye contact. This period was characterized by “intuitive hyper-vigilance,” in which parents perceived these signs as indications that “something was not right”:

“*We started to notice that something wasn't as we expected because he didn't respond when we called him”* (P1).

Participants noted that these initial suspicions were not always validated by healthcare professionals, who sometimes attributed the difficulties to developmental variations. These types of responses contributed to prolonging the period of uncertainty, during which time, parents continued to observe differences in their child's development without receiving a clear explanation:

“*The pediatrician said it was developmental delay”* (P4).

Diagnostic confirmation, often following a prolonged period of uncertainty, was experienced as a significant emotional turning point. Parents described an initial reaction characterized by shock, sadness and difficulty assimilating the implications of the diagnosis:

“*When they finally confirmed the diagnosis, it was a very hard blow”* (P8).

This initial impact coexisted, in some accounts, with a sense of relief derived from finally obtaining an explanation for the developmental differences they had previously observed. Relief did not eliminate distress; rather, both emotions could occur simultaneously, reflecting the ambivalent nature of the diagnostic experience.

The narratives suggested that grief and adjustment did not follow a uniform or linear sequence. Parents described moving between uncertainty, emotional pain, the search for explanations, concern about the future and a gradual reformulation of their expectations. The experience of grief was therefore associated not with the loss of the child, but with the need to reconsider previously imagined expectations regarding development, independence and family life:

“*Hearing the word autism completely changes how you see your child's future”* (P9).

When reflecting retrospectively on their experiences, some participants described a shift toward greater understanding and acceptance of their child's individual developmental trajectory. However, this adaptation did not necessarily imply the disappearance of sadness or uncertainty, as concerns could re-emerge in relation to new developmental stages and future support needs.

#### 3.1.2 Subtheme 1.2: Institutional disorientation after the diagnosis

After receiving the diagnosis, parents reported a widespread lack of guidance and institutional support. This moment was described as particularly critical from an emotional perspective.

“*You go home with the diagnosis and you say “Now what?”* (P11).

The lack of clear and structured information about resources, support and the steps to be taken generated uncertainty and left parents to seek help independently, often through contact with other families:

“*Nobody tells you what steps to take”* (P2).

“*You find out by talking to other families”* (P1).

Some participants retrospectively described earlier experiences in which greater parental initiative was required to access resources or diagnostic assessments.

“*Years ago, they gave you the diagnosis and then you were on your own”* (P15).

This process was perceived as a solitary search within a complex and inaccessible system.

#### 3.1.3 Subtheme 1.3: Transformation of parental expectations

When reflecting on their experiences, participants described changes in their expectations, including a shift from a focus on normalization toward greater acceptance of their child's individual developmental trajectory. This process involved a redefinition of achievements, objectives and goals related to their child's development. Parents also highlighted the importance of valuing progress that might be considered minor in other contexts:

“*You learn to value advances that seem small to others”* (P14).

“*Our dreams and expectations for his development have changed”* (P3).

A tendency to adjust expectations to adapt to the child's pace was identified, avoiding frustration for both the child and the parents:

“*We try not to set rigid expectations; we adapt from day to day”* (P8).

“*If he points to the pictogram to tell me he wants something, that's enough for me”* (P9).

These accounts suggest a process of adjustment through which parents reformulated expectations and placed greater value on functional progress and meaningful achievements in everyday life.

Across these subthemes, diagnostic confirmation emerged not only as the identification of a clinical condition but also as a reorganization of meaning. Parents retrospectively reinterpreted their earlier concerns and began to reconstruct previously imagined expectations regarding their child's development, independence and future family life.

### 3.2 Theme 2: Emotional impact, coping and reorganization of family life

#### 3.2.1 Subtheme 2.1: Intensification of the bond

Participants described a strengthening of their bond with their children, characterized by greater involvement in their care, a relationship of high dependence and protection, in response to their needs. This bond is built daily through constant presence and direct involvement in the child's routines, care, and development:

“*A different, stronger connection is formed with your child with ASD”* (P12).

“*It is a bond of protection and resilience”* (P15).

Parents assumed roles that extended beyond conventional parenting and caregiving, positioning themselves as mediators, advocates and facilitators of their child's development. Parents noted a perception of high dependency on the part of the child. They described how this relationship is built on observation, anticipating needs, and continuously adapting to the child's responses:

“*You feel that your child really needs you for everything”* (P2).

The narratives reflect a close and continuous relationship in which parents are central figures in the child's daily support. This relationship is characterized by continuity of care and direct involvement in the child's needs, forming a bond that the participants themselves describe as more intense and distinct compared to their experiences caring for other children.

#### 3.2.2 Subtheme 2.2: Reorganization of daily life

Parents described substantial changes in the organization of everyday family life during the diagnostic and post-diagnostic periods, including the detailed structuring of household activities and routines. This reorganization is reflected in the constant planning of time and the adaptation of family dynamics to the child's needs:

“*Our lives revolve largely around their highly structured daily routines”* (P9).

Participants said that routine plays a central role in day-to-day working, forming the basis upon which daily activities are organized. Habitual tasks, such as outings, leisure activities and family events, become conditioned by the predictability and stability necessary for the child:

“*Practically every afternoon is planned”* (P5).

The need to anticipate potentially destabilizing situations created a constant state of alert among parents.

“*We try to anticipate everything to prevent crises, and that means we are constantly on alert”* (P4).

The narratives reflect how this anticipation is not limited to central activities, but rather extends to multiple aspects of daily life, including schedules, travel and changes in the environment. Parents also reported reduced spontaneity and limitations in social participation, which contributed to a degree of family isolation.

#### 3.2.3 Subtheme 2.3: Impact on siblings and the family system

Participants described how the presence of a child with ASD influences the overall dynamics of the family system, with the siblings of children with ASD identified as a particularly affected group. Participants noted a tendency toward early maturation and the assumption of additional responsibilities. The impact of which is reflected in the distribution of time, attention and responsibilities within the home:

“*Sometimes siblings are the ones who are forgotten, and they suffer as well. We have less time for them because most of our time is devoted to caring for their sibling with ASD”* (P7).

Parents reported difficulties in distributing time and attention among family members because of the specific care and support needs of the child with ASD. This situation affects the organization of family time and the parents' availability to attend to other children. Parents expressed feelings of guilt about the unequal distribution of time and attention, also describing the need to create separate spaces for the siblings, with the goal of maintaining moments of individualized attention within the family dynamic:

“*We try to find time just for him, but it is often difficult to do so in everyday life”* (P9).

Likewise, the narratives reflect a persistent concern regarding the role siblings might assume in the future. This concern is linked to the continuity of care and the knowledge that siblings can acquire concerning the younger child's needs:

“*If we're ever gone, they'll have to know how to help their sister”* (P6).

These accounts show that the diagnosis affected the distribution of parental attention, the responsibilities attributed to family members and expectations regarding future care.

#### 3.2.4 Subtheme 2.4: Coping strategies and active family adaptation

Coping emerged across participants' accounts as a dynamic combination of flexible goal-setting, active resource-seeking and the gradual adaptation of expectations. Rather than pursuing predetermined developmental outcomes, some parents described maintaining an ongoing commitment to their child's progress while adjusting their goals according to the child's abilities and circumstances:

“*We will go as far as we can, and once we reach that point, we will continue working”* (P2).

Parents also described proactively mobilizing formal support to respond to their child's needs and reduce the demands placed on the family. This included seeking specialized services and organizing personal assistance across different times of the day:

“*We try to arrange as many hours of personal assistance as possible, in the morning and in the afternoon. In other words, we try to do everything we can”* (P7).

These strategies operated alongside other forms of coping identified across the preceding themes, including seeking informal guidance from other families, creating predictable routines, anticipating potentially distressing situations and progressively reformulating expectations. Together, these responses reflected practical, relational and meaning-oriented forms of coping through which parents attempted to balance their child's needs, everyday family functioning and concerns about the future.

However, these strategies were not experienced as uniformly protective. The continuous search for resources and the need to organize extensive support required sustained parental effort and reflected the limited guidance available from formal services. Coping therefore represented an evolving and context-dependent process rather than the definitive resolution of the emotional and practical demands associated with diagnosis.

Taken together, these accounts suggest that routines, anticipation, advocacy and flexible goal-setting were not merely practical responses to everyday demands. They also represented ways of restoring predictability and parental agency within a context experienced as uncertain and insufficiently supported.

### 3.3 Theme 3: Concern for the future

#### 3.3.1 Subtheme 3.1: Uncertainty regarding the future of care

Concern about the future emerged as a central element in the participants' discourse. This concern appeared constantly and across all aspects, especially regarding the continuity of care in the absence of the parents. This worry was repeatedly expressed throughout the interviews, establishing itself as a persistent concern in the family experience:

“*The biggest worry is what will happen when we're gone”* (P1).

Participants described this uncertainty as a central issue in their daily lives, tied in with the need to ensure that the child's needs can be understood and met by others in the future. In this sense, the narratives reflect parents' perception that they were the primary sources of understanding for their child, particularly regarding communication, routines and responses to everyday situations:

“*You feel like you're their only voice. You wonder who will interpret their needs when you're no longer there”* (P8).

Some participants expressed this concern in physical and emotional terms, describing bodily reactions associated with thoughts regarding the future. Uncertainty about future care is also linked to a lack of clarity about long-term resources and the need to anticipate possible scenarios:

“*Sometimes you think about the future and feel a tightness in your chest, because you know that society is not prepared to care for them as we do at home”* (P5).

This fear transcended logistical aspects, incorporating an emotional dimension that was seen as being linked to the child's potential vulnerability in the absence of their family environment. Participants reported ongoing uncertainty regarding the continuity of care, the understanding of the child's needs and the availability of support in the future.

#### 3.3.2 Subtheme 3.2: The independence paradox

Parents described their child's independence as an important goal in everyday life, associated with the progressive development of skills for greater autonomy. This aspiration appeared repeatedly among the participants, linked to the desire for the child to be able to function independently in different contexts:

“*We try to make sure that he's as independent as possible”* (P3).

“*We are concerned about their future employment and independence”* (P10).

However, participants pointed out that this goal coexists with the need to adapt expectations to the child's pace and abilities. Independence is not presented as a uniform objective here, but rather as a gradual and variable process:

“*Not setting fixed expectations, other than letting the child continue progressing to the level they are capable of”* (P6).

“*You learn to value progress that might seem small to others”* (P10).

Some participants described situations in which the attempt to promote certain learning could cause distress to the child.

“*Sometimes I think I might have focused too much, the child has to write. In the end, you keep adding bits and pieces and it can stress them out”* (P8).

These experiences reflect how parents continually observed and adjusted their expectations and demands according to their child's responses. Within this context, participants mentioned the importance of avoiding rigid expectations that may be difficult to meet.

“*If not, we can sometimes fall into the trap of trying to frustrate the child with our expectations. That scares me a lot”* (P3).

Participants described a process in which promoting autonomy was linked to the continuous adaptation of expectations, based on the child's abilities and responses and creating a dynamic balance within parents' efforts to promote autonomy.

#### 3.3.3 Subtheme 3.3: Limitations in the support system in adult life

Participants spoke of a persistent concern regarding the availability of resources and support after compulsory schooling. This anxiety appears to be linked to the perception of reduced services upon reaching adulthood:

“*The future scares me because it sometimes seems that, once they turn 18, they disappear from the administration's radar”* (P2).

Parents identified a marked difference between the support available during the educational stage and that available in adulthood. In this regard, they noted a lack of clarity regarding the options available after completing schooling. Participants also expressed concern about opportunities for inclusion in the workplace:

“*Where will they work? Who will give them a chance knowing that they need different timings?”* (P14).

Parents described the need to remain actively involved in seeking resources and support, both in the present and for the future.

“*We try to arrange as many hours of personal assistance as possible, in the morning and in the afternoon. In other words, we try to do everything we can”* (P7).

This effort is reflected in planning interventions, organizing support and seeking alternatives that allow for continuity of care. Some participants further highlighted the importance of accessing specialized resources at early stages to facilitate future development through therapies, educational support and intervention strategies.

“*We try to access the best resources now so that tomorrow will be less difficult.”* (P8).

Participants describe a perception of uncertainty regarding the support available in adult life, as well as the need for sustained parental involvement to secure continuity of support over time.

Underlying these concerns was not only uncertainty about the availability of future services, but also parents' fear that their child's needs might no longer be understood, interpreted or adequately supported in the absence of parental presence.

## 4 Discussion

The present study set out to explore the lived experiences and expectations of Spanish parents during their child's ASD diagnostic process, as well as their perceptions of the resulting reorganization of family life and future-oriented concerns. Parents' accounts suggest that an ASD diagnosis transcends clinical categorization and forms part of a complex family transition characterized by prolonged uncertainty, disruption of everyday life and gradual adaptation through advocacy and acceptance of neurodiversity.

Across the three themes, parents described a prolonged and uncertain diagnostic process, an ambivalent emotional response, the progressive reformulation of expectations, intensive organization of everyday routines, expanded parental roles, perceived effects on siblings and persistent concerns about future support. Together, these findings suggest that diagnostic confirmation was experienced not as an isolated clinical event but as part of a broader reorganization of everyday family life. The discussion is therefore organized around the three empirically derived areas represented in the Results: the diagnostic process and parental adjustment; coping and the reorganization of family life; and future-oriented concerns. Theoretical and empirical literature is used to contextualize these findings rather than as evidence of additional themes identified in the present study.

When contextualizing our results with recent literature, it can be seen that the burden of daily care and the barriers encountered across healthcare, educational and social-care systems closely converge with those documented in other severe neuro-developmental conditions in childhood. The diagnostic process, the burden of daily care, and structural and healthcare-system barriers profoundly impact not only the patient but also the entire family dynamic and the parents' mental health (García-Bravo et al., 2023; Garcia-Bravo et al., 2025; Mackley et al., 2018; Crellin et al., 2023; Willmen et al., 2021; Jeffrey et al., 2021). Integrating these perspectives allows us to define the main systemic challenges and the strategies needed to improve coordination across healthcare, educational and social-care services.

Those taking part in this study described a ‘diagnostic odyssey' in which early parental suspicions frequently clashed with minimization or invalidation by healthcare professionals, especially in primary care. This intuitive, non-validated hyper-vigilance generates a period of ambiguity that delays access to early intervention. These results are consistent with those highlighted by García-Bravo et al. (2023)), as they document the tensions between professionals and parents. The authors note that the lack of active listening during initial consultations not only prolongs the time to diagnosis but also undermines families' trust in the healthcare system.

Participants' reports of minimization and delayed recognition are consistent with previous research identifying system-level barriers to timely ASD assessment, including limited specialist availability and insufficient training among primary care professionals (Mathur et al., 2026). These specific system-level factors were not examined directly in the present study and are therefore used to contextualize, rather than extend, participants' accounts. More directly supported by the findings is the need for clear and paced communication at diagnostic confirmation, explicit information about subsequent steps and accessible post-diagnostic referral pathways. Given the shock and institutional disorientation described by participants, emotional support should also be available to parents rather than restricting post-diagnostic care to the child alone (Daniels et al., 2021; García-Bravo et al., 2023).

Diagnostic confirmation was described by participants as a profoundly ambivalent event, in which shock and sadness coexisted with relief at finally obtaining an explanation for their child's developmental differences. These accounts are consistent with the transition from “parent” to “expert” described by Edwards et al. (2018)). Faced with institutional disorientation and the absence of a clear post-diagnostic pathway, parents assumed an increasingly active role in seeking interventions, accessing support and advocating for their children, while gradually reformulating their previous expectations regarding development and family life.

Because the year or date of diagnosis was not collected, time since diagnosis could not be examined as a contextual or comparative variable. The study therefore cannot determine whether variation in participants' accounts was associated with elapsed time since diagnosis, the child's age or support needs, family circumstances or experiences with services. Nevertheless, both the recurrent concerns described by participants and previous research caution against assuming a simple linear relationship between elapsed time and grief resolution, as uncertainty and feelings of loss may re-emerge during later developmental and service transitions (Bravo-Benítez et al., 2019; Coughlin and Sethares, 2017; Mihandoust et al., 2021). In this respect, participants' experiences resemble ambiguous loss and chronic sorrow: the child remains present and valued, while parents repeatedly reformulate previously imagined expectations concerning development, independence and family life (Boss, 1999; O'Brien, 2007; Coughlin and Sethares, 2017). Adaptation should therefore not be understood as the definitive resolution of grief, but as an evolving capacity to integrate the diagnosis into family life while responding to new challenges and transitions. These findings support the need for post-diagnostic services that do not limit emotional support to the moment in which the diagnosis is communicated. Professionals should recognize that parents may require different forms of information, validation and psychological support at successive developmental transitions, particularly when concerns about schooling, independence, adulthood and continuity of care re-emerge.

Although participants described diagnostic confirmation as an important turning point, caregiving did not begin with the diagnosis. During the pre-diagnostic period, parents were already closely observing developmental differences, seeking explanations and repeatedly advocating for professional recognition of their concerns, a trajectory also identified in qualitative syntheses of parents' experiences of the autism diagnostic process (Boshoff et al., 2019; Smith-Young et al., 2025). In their retrospective accounts, participants described these efforts as becoming more structured and future-oriented following diagnostic confirmation, involving the selection of interventions, navigation of services, coordination of support, organization of routines, advocacy and the gradual recalibration of expectations. This evolution is consistent with Edwards et al. (2018)), who found that parental decision-making developed over time as parents moved from being a “parent” to becoming an “expert” in their child's needs. Diagnostic confirmation may therefore be better understood as reframing and intensifying pre-existing caregiving responsibilities rather than creating an entirely new pattern of care. Nevertheless, because participants' accounts were retrospective and the study was not designed as a longitudinal or matched pre–post comparison, this interpretation should be considered exploratory rather than causal.

Similar experiences of diagnostic uncertainty, repeated parental advocacy and ambivalent emotional responses have been described in qualitative studies involving parents of children with other pediatric neurodevelopmental conditions (García-Bravo et al., 2023; Garcia-Bravo et al., 2025). These parallels help to contextualize the present findings within broader challenges associated with diagnostic navigation and post-diagnostic support. However, they should not be interpreted as indicating that the experiences, clinical trajectories or support needs associated with these conditions are equivalent.

Parents' accounts indicate that raising a child with ASD can substantially reorganize family life and relationships. Our findings regarding highly structured routines, intensive parental involvement and the impact on siblings are consistent with evidence indicating elevated levels of depression, chronic stress and anxiety among parents and caregivers (Sánchez Amate and Luque de la Rosa, 2024). Professionals have also noted that, in some cultural contexts, spiritual beliefs or stigma associated with neurodevelopmental conditions may contribute to social isolation or delay the search for support. At the same time, exposure to contradictory information through social media and artificial intelligence may increase confusion and psychosocial stress, particularly among parents with higher levels of education (Lotfi et al., 2026).

Viewed through a family systems perspective, the reorganization described by participants extended beyond changes in routines and caregiving demands. Parents perceived that the diagnostic process and their child's ongoing support needs affected several interdependent relationships within the household, including the parent–child relationship, parental roles and sibling relationships. Parents expanded their functions to become advocates, interpreters of their child's needs and coordinators of support, thereby reducing the distinction between conventional parenting and care-management responsibilities. At the same time, the concentration of time and attention on the child with ASD affected siblings, who were perceived by parents as receiving less individual attention, maturing earlier and, in some cases, potentially assuming future support responsibilities. These accounts can be interpreted as illustrating how changes affecting one family member may be associated with adaptations across the wider family system and its different subsystems (Cox and Paley, 1997; Rogers et al., 2022).

The findings also reveal a tension between parents' pre-existing expectations and the roles and family trajectories that emerged following diagnosis. Parents reformulated expectations not only regarding their child's development and independence but also regarding family participation, sibling relationships and future care arrangements. Their attempts to preserve individualized time for siblings suggest an active effort to balance competing needs within the household. Nevertheless, concerns that siblings might eventually assume caregiving responsibilities indicate that family roles and boundaries remain subject to continued negotiation across the life course. This interpretation is consistent with contemporary systemic approaches emphasizing that family functioning arises from dynamic interactions among individual, relational, developmental and sociocultural processes rather than from the experience of a single family member in isolation (Rogers et al., 2022).

However, this systemic interpretation should be considered in light of the study's unit of analysis. The findings derive from individual parents' accounts rather than from dyadic or whole-family narratives. Therefore, the reported changes in family roles, sibling relationships, routines and expectations represent parents' perceptions of family life and should not be interpreted as a formal family-level analysis or as the collectively shared experience of all household members. The study was unable to examine agreement, disagreement or variation between mothers, fathers, siblings and other family members.

It is essential to emphasize the feminisation and gender bias inherent in this care. As the literature suggests, mothers predominantly assume the role of primary caregiver, which frequently leads them to cut their working hours or abandon their professional careers, reducing their financial independence and social network (Muiño Tato, 2026). However, variables such as adaptive coping (through positive reappraisal) and dyadic adjustment (the quality of the couple's relationship) emerge as crucial protective factors capable of mitigating emotional overload and improving the quality of family life (García-Bravo et al., 2023; Muiño Tato, 2026).

Participants' reports of seeking services, coordinating support, advocating for their children and adapting developmental goals are consistent with the transition from “parent” to “expert” described by Edwards et al. (2018)). In the present study, this transformation was reflected in parents' growing experiential knowledge, their active navigation of fragmented services and their continuous adjustment of expectations to their child's abilities and responses. This process was closely connected to the tension between promoting autonomy and avoiding rigid or potentially distressing demands.

Beyond the emotional impact of diagnosis, our findings identified several interrelated coping strategies. Parents sought informal support from other families, structured routines to increase predictability, anticipated situations that might trigger distress, actively searched for services and gradually reformulated their expectations according to their child's individual developmental trajectory. These responses combined relational, practical and meaning-oriented forms of coping. In particular, peer support and the positive reappraisal of previously imagined expectations are consistent with the adaptive strategies highlighted by (Sánchez Amate and Luque de la Rosa 2024), who emphasize the potential protective role of social support and positive reappraisal in family well-being. However, these strategies should not be understood as uniformly protective or as evidence that the demands associated with care had been resolved. Although routines and anticipation helped parents create greater stability in everyday family life, they also required continuous vigilance and reduced spontaneity. Similarly, active advocacy and resource-seeking enabled parents to access support but often emerged as a response to fragmented institutional guidance. Coping therefore constituted a dynamic and context-dependent process through which parents attempted to balance their child's needs, overall family functioning and their own evolving expectations.

Future-oriented concerns constituted a distinct finding rather than merely an extension of present caregiving demands. Parents' fears centered on whether their child's communication and support needs would continue to be understood in their absence, whether services would remain available after compulsory education and whether meaningful opportunities for autonomy and employment would be accessible. These accounts support the need for transition planning that begins before the end of schooling and coordinates educational, healthcare, social-care and employment-related services across adolescence and adulthood. Nevertheless, the study did not assess the actual availability, accessibility or effectiveness of specific adult services. The findings therefore represent parents' perceptions of uncertainty and discontinuity rather than an objective evaluation of the Spanish support system (Autismo España, 2024; Garcia-Bravo et al., 2025).

### 4.1 Limitations and future lines of research

Although this study provides in-depth insight into parents' lived experiences and their perceptions of the impact of an ASD diagnosis on family life, several limitations should be acknowledged. The purposive recruitment of participants within the Spanish context and the predominance of mothers may limit the transferability of the findings to other sociocultural settings and family configurations. The small number of fathers prevented any meaningful comparison of maternal and paternal accounts; therefore, parent gender was reported descriptively but was not used as an analytical grouping variable. In addition, the retrospective nature of participants' accounts makes the findings susceptible to recall bias.

The year or date of diagnosis was not collected as a standardized participant characteristic; therefore, time since diagnosis could neither be described for the sample nor examined as a contextual or comparative variable. The study also did not systematically elicit matched descriptions of caregiving practices before and after diagnostic confirmation. Consequently, participants' retrospective accounts do not permit temporal or causal attribution of changes in parental roles, family routines, coping strategies or support-seeking practices to diagnostic confirmation alone. Nor can the study determine whether variation among participants' accounts was associated with elapsed time since diagnosis rather than with the child's age, support needs, family circumstances or experiences with services. Diagnostic confirmation may have reframed or intensified caregiving responsibilities that had already emerged during the pre-diagnostic period, but the cross-sectional design does not allow the direction, magnitude or temporal evolution of these changes to be established. Accordingly, the findings should not be interpreted as a comparative reconstruction of family functioning before and after diagnosis or as evidence of a uniform chronological transition.

The individual parent, rather than the family as a whole, was the unit of participation and analysis. As no dyadic or whole-family interviews were conducted, the findings reflect individual parents' accounts of the diagnostic process and their perceptions of its consequences for family life. The perspectives of partners, siblings and other caregivers were not obtained directly, and agreement, disagreement or variation among members of the same household could not be examined. Accordingly, although the findings were interpreted through a family systems perspective in the Discussion, they should not be understood as representing a formal family-level or dyadic analysis. In particular, findings concerning siblings' experiences and anticipated responsibilities reflect parental perceptions rather than siblings' own accounts.

Future research should recruit participants from more diverse socioeconomic and cultural backgrounds and include a wider range of family configurations. Future cross-sectional studies should systematically collect and report the date or year of diagnosis and, where relevant, purposively sample parents at different points following diagnostic confirmation to examine how accounts may vary across temporal contexts. Longitudinal qualitative studies beginning during the pre-diagnostic period and involving repeated data collection would help clarify how parental roles, caregiving practices, family routines, coping strategies and support needs evolve throughout the diagnostic trajectory (Audulv et al., 2022). Dyadic and multi-informant designs involving mothers, fathers, siblings and other significant caregivers would also enable researchers to examine how experiences, expectations and responsibilities are negotiated among different members of the family system. Mixed-method approaches could further integrate individual narratives with longitudinal measures of parental well-being, family functioning and support needs, contributing to a more comprehensive, equitable and family-centered understanding of neurodevelopmental care.

## 5 Conclusions

Parents' accounts suggest that an ASD diagnosis can profoundly reshape everyday family life. The diagnostic process was frequently experienced as a difficult transition marked by prolonged uncertainty, limited validation of parents' initial concerns and delays in accessing appropriate support for the child.

In the absence of adequate post-diagnostic guidance, parents described a substantial expansion of their roles, moving beyond conventional parenting responsibilities to become advocates, coordinators and experts in their child's needs. Although this transition could strengthen the parent–child bond and support active adaptation, it also required intensive organization of routines, affected the distribution of parental time and attention among siblings and limited opportunities for family spontaneity and social participation.

Uncertainty regarding future autonomy, continuity of care and the availability of services in adulthood emerged as a central concern among participating parents. These findings support the need for policies and services that address not only the needs of the child with ASD but also parents' emotional wellbeing and the functioning of the wider family system. Healthcare, educational and social-care services should provide coordinated and sustained support throughout the diagnostic process and across subsequent developmental transitions.

## Data Availability

The raw data supporting the conclusions of this article will be made available by the authors, without undue reservation.
