# Correction: “Listening to the word autism changes everything”: the impact of an Autism Spectrum disorder diagnosis on families

**DOI:** 10.3389/fpsyg.2026.2002051

**Published:** 2026-10-01

**Authors:** M Gloria Gallego-Jiménez, Sofía Torrecilla-Manresa, Cristina García-Bravo

**Affiliations:** 1 Faculty of Humanities and Communication, Department of Education. CEU San Pablo University, Madrid, Spain; 2 Department of Physical Therapy, Occupational Therapy, Rehabilitation and Physical Medicine, Research Group of Humanities and Qualitative Research in Health Science, Universidad Rey Juan Carlos, Alcorcon, Spain

**Keywords:** Autism Spectrum disorder, diagnosis, lived experience family life, parents, qualitative study

**Funding** for this article was erroneously omitted. The statement appears below:

The author(s) declared that financial support was received for this work and/or its publication. The project was funded by the Research Projects Program of CEU San Pablo University within the framework of institutional projects (reference MPF 124GG/1): Influence of teachers' beliefs on cognitive performance and executive functions in students with syndromic and idiopathic ASD. This research received no external funding. The present text was developed within the framework of a Research and Innovation Group (GIR) at Universidad San Pablo CEU, titled Learning, Neurodidactics, Personalized and Inclusive Education (ANEPI) https://www.uspceu.com/investigacion/grupos-investigacion/id/anepi (website accessed on October 22, 2025).

An **Acknowledgments** statement was erroneously omitted from the published article. The statement appears below:

The authors are grateful for the support of Universidad San Pablo CEU, and more specifically to the ANEPI Research Group (G24/3-13) and the DocenTEA Project (MPFI24GG). The authors would like to express their sincere gratitude to all the teachers who participated in this study, as well as to the experts who contributed as judges in the evaluation process.

The original version of this article has been updated.

